# Parasite intensity drives fetal development and sex allocation in a wild ungulate

**DOI:** 10.1038/s41598-020-72376-x

**Published:** 2020-09-24

**Authors:** O. Alejandro Aleuy, Emmanuel Serrano, Kathreen E. Ruckstuhl, Eric P. Hoberg, Susan Kutz

**Affiliations:** 1 Department of Biological Sciences, University of Calgary, 3330 Hospital Drive, Health Science Centre 2559, Calgary, AB Canada; 2 Department of Ecosystem and Public Health, Faculty of Veterinary Medicine, University of Calgary, Calgary, Canada; 3 Wildlife Ecology and Health Group (WE&H) and Servei d’Ecopatologia de Fauna Salvatge (SEFaS), Departament de Medicina i Cirurgia Animals, Universitat Autònoma de Barcelona, Bellaterra, Barcelona, Spain; 4 Dipartimento di Scienze Veterinarie, Universitá di Torino, Grugliasco, Turin, Italy; 5 Museum of Southwestern Biology and Department of Biology, University of New Mexico, Albuquerque, NM USA

**Keywords:** Ecological epidemiology, Population dynamics, Theoretical ecology, Sexual selection

## Abstract

An understanding of the mechanisms influencing prenatal characteristics is fundamental to comprehend the role of ecological and evolutionary processes behind survival and reproductive success in animals. Although the negative influence of parasites on host fitness is undisputable, we know very little about how parasitic infection in reproductive females might influence prenatal factors such as fetal development and sex allocation. Using an archival collection of Dall’s sheep (*Ovis dalli dalli*), a capital breeder that depends on its body reserves to overcome the arctic winter, we investigated the direct and indirect impacts of the parasite community on fetal development and sex allocation. Using partial least squares modelling, we observed a negative effect of parasite community on fetal development, driven primarily by the nematode *Marshallagia marshalli*. Principal component analysis demonstrated that mothers with low parasite burden and in good body condition were more likely to have female versus male fetuses. This association was primarily driven by the indirect effect of *M. marshalli* on ewe body condition. Refining our knowledge of the direct and indirect impact that parasite communities can have on reproduction in mammals is critical for understanding the effects of infectious diseases on wildlife populations. This can be particularly relevant for species living in ecosystems sensitive to the effects of global climate change.

## Introduction

Parasites can negatively affect pregnancy rate^1–3^, offspring survival^4–6^, and offspring size^7^. However, very little is known about how prenatal characteristics, such as fetal development and sex allocation, can be influenced by parasitic infections in the mother. Determining the factors that affect prenatal characteristics is key to understanding the ecological and evolutionary mechanisms behind survival and reproductive success in animals^8,9^. For instance, fetal development has direct consequences for postnatal physiology, metabolism, growth and immune response (e.g. birds^9,10^, people^11,12^, wild mammals^13^, and various domestic species^14–19^), while sex allocation can influence population dynamics through a variety of sex-biased mechanisms (e.g. sex-biased mortality and sex-biased reproduction rate)^20^.

Parasites can affect fetus development through several pathways including (i) a direct effect of parasite virulence on fetal development or survival causing pregnancy failure (e.g. abortion, fetal mummification, fetal reabsorption), as seen with bacteria such as *Brucella abortus* and protozoa such as *Neospora caninum*^21,22^, (ii) an indirect effect of the energetic cost of parasitic infection on the mother (e.g. parasite nutrition, host immune response, appetite depression), resulting in decreased nutrition to the fetus and a developmental delay^23,24^, and (iii) an indirect effect of parasites interrupting or delaying pregnancy due to a negative effect on host condition resulting in delayed ovulation and conception, which can translate to smaller fetus size^25^. The few attempts to quantify the effects of parasites on fetal development have focused on humans, where the infection with gastrointestinal helminths can cause delayed fetal growth and premature parturition (reviewed in^26^).

There is theoretical and empirical evidence supporting the hypothesis that sex allocation can be directly influenced by extrauterine factors^27^. The Trivers-Willard hypothesis^28^, for instance, suggests that in polygynous mating systems, mothers in good condition will produce more sons than daughters because they can afford to provide the needed maternal care to produce a high-quality son. This strategy yields the greatest fitness return in species where variance in reproductive success is strongly correlated with body size in males but not in females. Conversely, the Local Resource Competition hypothesis suggests that females in poor condition will produce more sons because sons will disperse, and thus are less likely to compete with the mother for resources during adulthood^29^. Many efforts have been made to understand the physiological mechanisms behind these sex-biased trends and their relationship with different factors, including mother condition and stress (e.g.^30–34^.). Although the role of mother’s parasitic infection on sex allocation has not been documented in wild mammals, studies investigating the influence of protozoan parasite, *Toxoplasma gondii*, in sex allocation of people and mice have demonstrated an important link between this parasite and sex adjustments^35–37^.

We took advantage of an unprecedented historical collection of parasites and associated data in Dall’s sheep (*Ovis dalli dalli*, see Fig. 2A) collected from the Mackenzie Mountains, Canada in 1971–1972^38,39^, to assess the effect of gastrointestinal parasites on fetal development and sex allocation. Dall’s sheep are highly philopatric, females normally remain in the same general area their entire lives, whereas subadult males disperse from their mothers’ group to join bachelor groups^40^. Also, these free-ranging wild sheep take advantage of summer pastures to fuel their body reserves in anticipation of winter shortages and, as a capital breeder, use the stored energy to support the cost of reproduction during winter^41–43^. Under the current conditions of accelerating global warming, seasonal patterns of climate and vegetation growth are being altered in Alpine and Arctic ecosystems worldwide^44^. Concurrently, patterns of parasitism are changing, with amplification and range expansion of some parasitic species already demonstrated at higher latitudes^45–47^. These changes shape body condition, survival, and reproductive patterns of ungulates living in these environments^48,49^. Yet the synergistic effects of parasitism on body reserves, and ultimately on the reproductive outcomes (fetal development and sex allocation), might go unnoticed.

We had two main research objectives: (i) to determine the association of fetal development with the mother’s age, body condition, and infection intensity with gastrointestinal parasites, and (ii) to determine the association of fetus sex allocation with the same extrauterine factors. We hypothesized that, due to the high energetic costs of gastrointestinal parasites to their host, they are an important extrauterine factor determining fetal characteristics in Dall’s sheep. We predicted that, due to their negative impact on host condition, higher parasite burdens would be associated with smaller and lighter fetuses and a higher probability of having daughters than sons. To test this, we used a combination of Partial Least Square Path Modeling approach, an innovative regression procedure used to study causal relationships based on observational data, and traditional regression approaches.

## Materials and methods

### Sample collection

#### Dall’s sheep sampling and data collection

Scientific collections of wild Dall’s sheep occurred between February 17–19, 1971, and February 17–22, 1972, in the Mackenzie Mountains, Northwest Territories (NWT), Canada. This work was led by Dr. Norman Simmons with the Canadian Wildlife Service, with the primary objective of doing a demographic study of sheep^38^. The parasite specimens and the associated data collected from these sheep were located in the spring of 2000 at the Canadian Museum of Nature (CMN) in Ottawa (Ontario, Canada) and are permanently archived as “The Simmons Collection”^39^.

The Simmons Collection contained information from 70 pregnant ewes. The information documented from each ewe included: age in years based on tooth cementum annuli; body weight (kg); body length and chest girth, the species and number of adult helminth parasites in the abomasum, small intestine, large intestine and caecum and; the sex, weight (kg) and total body length (cm) of the fetus. Summary statistics of these variables between 1971 and 1972 can be found in Supplementary Table S1.

### Data analysis

#### Fetus development

We used Partial Least Square Path Modeling (PLS-PM), also known as “soft modelling” due to the liberal distribution assumptions and sample size requirements, to explore the association of gastrointestinal helminths and ewe characteristics (i.e. age and body condition) with fetus length and weight (used as a proxy for fetus development and/or fetus age), in Dall’s sheep. The PLS-PM approach is the intersection of Regression Models, Structural Equations Models, and Multiple Table Analysis^50,51^. Briefly, this approach quantifies the network relationship between a set of unobservable latent variables (LV) and a set of manifest variables (MV, i.e. parameters directly measured in the field or in the laboratory). The LVs are conceptual variables defined by one or several MVs and organized in a network of relationships where the connections among LVs are assumed to represent a cause-effect process. This network of relationships among LVs forms the inner model (also called structural model) while each group of MVs linked to a LV represents the outer, or measurement, model. The links among LV are quantified through path coefficients while the links between LV and MV are quantified through weights^52^.

**Figure 1:**
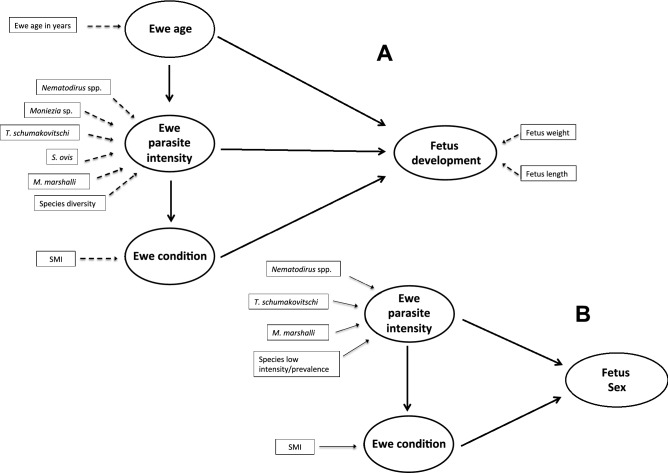
Initial path models describing phenotypical characteristics of Dall’s sheep fetuses collected in the Mackenzie Mountains, Canada during 1971 and 1972. (**A**) Path model for intrauterine fetal development. (**B**) Path model for fetus sex. Figure created using Microsoft PowerPoint, Version 14.1.

The analyses included ten MVs organized in the following three LVs (Fig. 1A); (i) age of mother in years, (ii) gastrointestinal parasites of the mother, defined by the intensity of each helminth species with prevalences higher than 10% and by the diversity of gastrointestinal parasite species (i.e. number of species infecting each ewe), and iii) ewe body condition, defined by a scale mass index (SMI) calculated for each ewe following Peig and Green (2009)^53^. The SMI is a versatile index due to its independence from body size and because it can be used to compare individuals from different populations^54^. The morphometric used to calculate the SMI was the ewe’s chest girth, as girth had the highest correlation with body weight (r = 0.69, *p* < 0.001, n = 67) among all the parameters available. After fitting the first model including all the variables, a model simplification was performed by removing those MVs uncorrelated with their own LVs. The PLS-PM approach does not depend on any distributional assumptions, therefore, a resampling procedure or bootstrap validation was used to get confidence intervals for evaluating the precision of the PLS parameter estimates (e.g. path coefficients, total effects and fit indices such as R^2^).

#### Fetus sex

The relationship between fetus sex and extrauterine characteristics in the ewe was investigated in three steps. First, a univariate comparison was performed to determine differences in sex distribution of the fetuses (chi-squared test) and differences in fetus morphometrics (i.e. weight and length; t-tests for differences in means, under the assumption of a normal distribution of means). Second, we performed a principal component analysis (PCA), including not only the burden of the most common parasite species but also other variables potentially linked to the sex of the fetus (e.g. age of the mother, or body condition of the mother). Then, we evaluated whether the values in the linear combination with the maximum variance (first PCA dimension) differed between female and male fetuses. The SMI index was used as a proxy for body condition of females and the log intensity of each parasite species with prevalence > 10% and/or median intensity of > 10 worms (i.e. *M. marshalli*, *Nematodirus* spp., and *Trichuris* sp.), as parasite burden. We also included the addition of the intensity of all parasite species with prevalence < 10% and/or median intensity < 10 worms (i.e. intensity of *Moniezia* sp. + *Trichostrongylus* sp. + *Skrjabinema ovis*) in the PCA analysis. Third, to explore indirect effects of parasites on fetus sex, a PLS-PM analysis was performed including body condition, parasite intensity of the mother (i.e. *M. marshalli*, *Nematodirus* spp., and *Trichuris* sp., and parasites with low intensity/prevalence), and fetus sex as LVs (Fig. 1B). After fitting the first model that included all the variables, a model simplification was performed by removing those MVs uncorrelated with their own LVs. The significant effects observed in the final PLS-PM were confirmed using Generalized Linear Models to fit models among the variables included in each significant effect. All the analyses were performed using R (Version 3.5.2) (R Core Team, 2013. R: A language and environment for statistical computing. R foundation for statistical computing Vienna, Austria) and using the “plspm”^55^ and “FactoMineR”^56^ packages.

## Results

**Figure 2:**
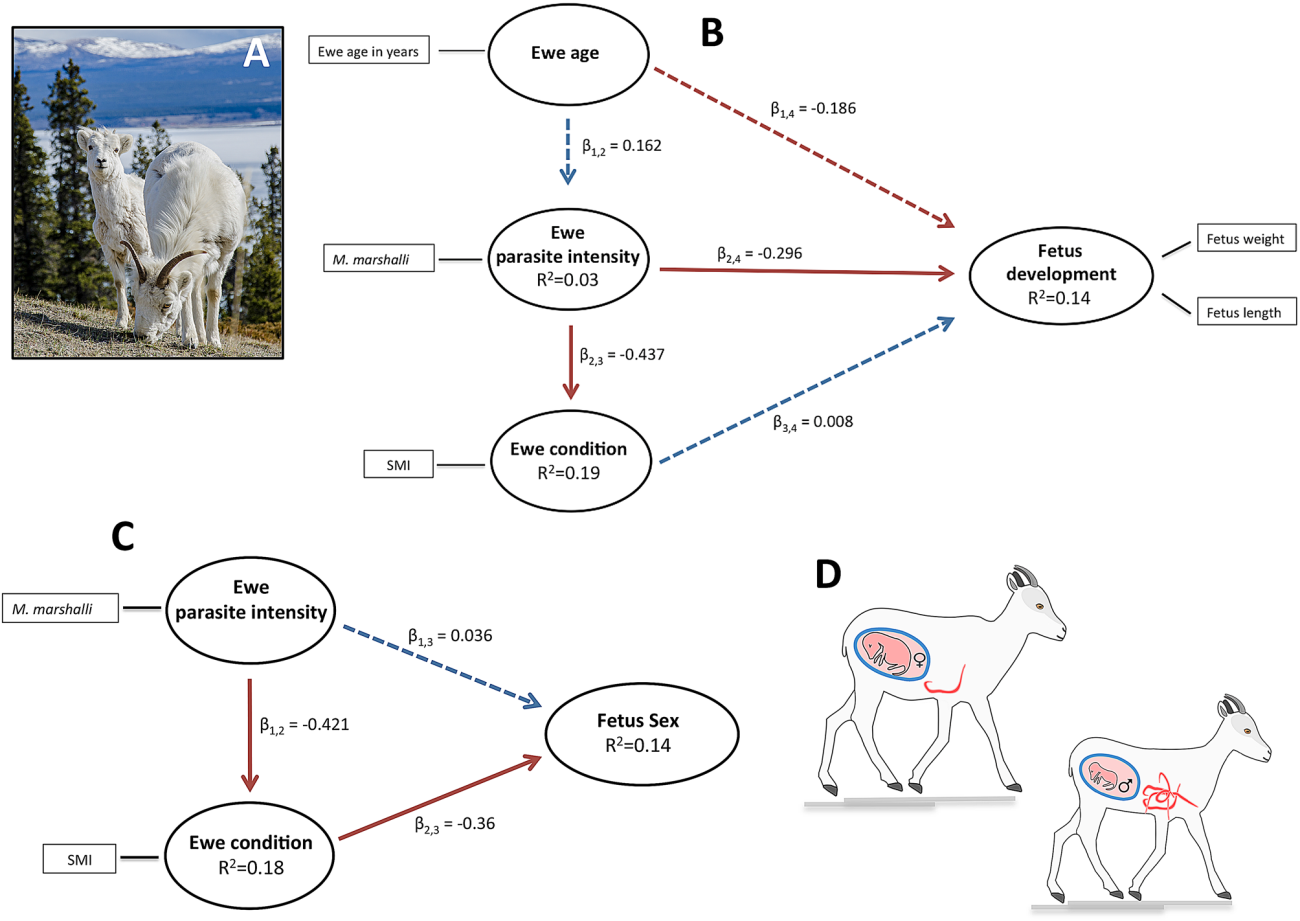
(**A**) Dall's sheep ewe and lamb. Final path model describing, (**B**) the fetal development and (**C**) sex allocation of Dall’s sheep. Blue and red arrows represent positive and negative associations between latent variables (LV). Solid and dashed arrows indicate significant and non-significant associations among LVs determined by bootstrapping validation. The R^2^ are coefficients of determination for each latent variable and indicate the amount of variance explained by their independent latent variables. β represents the path coefficient between LV (e.g. β1-2 path coefficient between LV 1 “Ewe age” and LV 2 “Ewe parasite intensity (**D**) summarizes the results from (**B**) and (**C**). Ewes in better condition and with low intensity of *M. marshalli* carry more female fetuses and those fetuses are larger than in ewes in low body condition and with high *M. marshalli* intensity. Ewes with low body condition and high *M. marshalli* intensity carry more male fetuses and their fetuses are smaller. Figure created using Microsoft PowerPoint, Version 14.1.

Descriptive statistics for the variables used in the full PLS-PM to investigate fetus development are in Table 1. The goodness of fit for the final PLS-PM was 0.31 and explained 14.2% (R^2^) of the observed fetal development variability. Parasite intensity of ewes (i.e. *M. marshalli* intensity) was negatively associated with fetus development, explaining the variation of this LV to a greater extent (68%) than mother’s age (30.6%) and mother’s body condition (0.8%) (Fig. 2B, Table 2). The correlation of each parasite species with its LV in the full model (Fig. 1A) was very low (λ < 0.3) with the exception of *M. marshalli* (λ = 0.86), therefore, *M. marshalli* was the only parasite species retained in the final model (Fig. 2B) (Supplementary Table S2). Although ewe body condition was negatively associated with the LV parasite intensity, there was no significant association between the ewe body condition and fetus development. The bootstrap results indicated that all relevant path coefficients, total effects and fit indices (R2) in the final model were significantly non-random at the ≤ 5% level (Table 3).

**Table 1:** Latent variables and descriptive statistics of the manifest variables used for fitting the causal model for fetus development and sex allocation of Dall’s sheep from the Mackenzie Mountains, Canada.

Latent variables	Manifest Variables	n	Descriptive statistics x̅ or median, (range) Prevalence (%), confidence interval (CI)
**Ewe age**			
	Age (years)	70	Median = 5.8 (1.75- 11.75)
**Ewe parasite intensity**			
	*Nematodirus* spp.	68	Median = 59 (1–1,158)
			92.3 (82.9–97.3)
	*Moniezia* sp.	68	Median = 1 (1–3)
			13.2 (6.6–24.1)
	*T. schumakovitschi*	68	Median = 13.5 (1–89)
			85.3 (74.1–92.3)
	*S. ovis*	68	Median = 5.5 (1–45)
			64.7 (52.1–75.6)
	*M. marshalli*	50	Median = 207 (42–2,106)
			100 (91.1–100)
	Species diversity	50	Median = 4 (2–5)
**Ewe condition**			
	Scale Mass Index (SMI)	70	x̅ = 51.1 (37.2–60.21)
**Fetus development**			
	Weight (gr)	70	x̅ = 235.0 (11.4–574.9)
	Total length (cm)	69	x̅ = 24.4 (10.5–33.7)

Median, Median parasite intensity calculated considering only parasite counts equal or higher than 1.

x̅, mean calculated using all the values.

min, minimum value. Not considering null counts.

max, maximum value.

**Table 2:** Regression coefficients and contribution (%) of each latent variable to the explained variability observed in the PLS-PM model describing fetus development of Dall’s sheep.

Explanatory variables for **Fetus development**	*β*	Correlation	Contribution to R2 (%)
Ewe parasite intensity	− 0.296	− 0.329	68.6
Ewe age	− 0.186	− 0.233	30.6
Ewe condition	0.008	0.135	0.8

β = path coefficient estimated by bootstrapping.

**Table 3:** Direct, indirect and total effects among latent variables (LV) in the final PLS-PM describing the development of Dall’s sheep fetuses (n = 49).

Relationship	Effects			95% CI
	Direct	Indirect	Total	
Ewe age—> Parasites	0.162	0	0.162	− 0.04–0.35
Ewe age—> Ewe condition	0	− 0.071	− 0.071	− 0.16–0.01
Ewe age—> Fetal development	− 0.186	− 0.048	− 0.234	− 0.5–− 0.04*
Parasites—> Ewe condition	− 0.437	0	− 0.437	− 0.66–− 0.08*
Parasites—> Fetal development	− 0.296	− 0.004	− 0.3	− 0.54–− 0.06*
Ewe condition—> Fetal development	0.009	0	0.009	− 0.36–0.4

The 95% confidence intervals were determined by bootstrapping validation.

**Figure 3:**
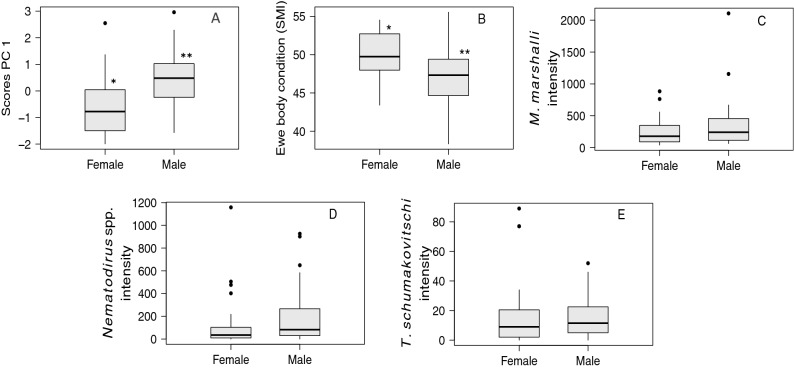
Comparison between male and female fetuses of Dall’s sheep. (**A**) Scores of the first component from the PCA analysis to determine the association between extra uterine characteristics and fetus sex in Dall’s sheep ewe. (**B**)–(**E**) Variables included in the first component of the same analysis. Significant differences are indicated with different number of * (p < 0.05). Figure created using R (Version 3.5.2) (R Core Team, 2013. R: A language and environment for statistical computing).

The sex of the fetuses were evenly distributed with 37 females and 33 males (Chi-squared, *X*^*2*^ = 0.36, df = 1, *p* value = 0.547). The weights for female (mean = 246.31 g, SE = 20.23) and male (mean = 222.29g, SE = 16.69) fetuses did not differ significantly (t-test, weight, t _0.91, 66_, *p* value = 0.363), nor did the total length of fetuses differ among sexes (Females mean = 24.56 cm, SE = 0.79; Males mean = 24.15 cm, SE = 0.67, t _0.39, 65_, *p* value = 0.693). The infection intensity of almost all parasite species was higher in ewes carrying a male fetus than in ewes with a female fetus. This trend was particularly evident for *M. marshalli*, *N. archari*, all *Nematodirus* spp. together, and all gastrointestinal helminths together (Supplementary Figure S1). The first two principal components (PC) explained 47.7% of the PCA variance. The scores of the first component were significantly different between male and female fetuses (R^2^ = 16%, *p* = 0.004, Fig. 3). The first PC was significantly associated with, in decreasing values of importance (loading), *M. marshalli* intensity (loading = 0.76), mother body condition (loading = − 0.72), *Nematodirus* spp. intensity (loading = 0.47), and *Trichuris* sp. intensity (loading = 0.29). The final PLS-PM model explained 14.2% (R^2^) of the variability observed in fetus sex, showing that ewes in good body condition and low parasite intensity were more likely to have female fetuses. A significant negative effect of parasites on ewe body condition was also observed (Table 4, Fig. 2C and Supplementary Table S3). In the final model, LV parasites only included *M. marshalli* (Fig. 2C).

**Table 4:** Direct, indirect and total effects among latent variables (LV) in the final PLS-PM describing fetus sex in Dall’s sheep (n = 50).

Relationship	Effects			95% CI
	Direct	Indirect	Total	
Parasites—> Ewes condition	− 0.421	0	− 0.421	− 0.614–− 0.0003*
Parasites—> Fetus Sex	0.036	0.152	0.187	− 0.04–0.385
Ewe condition—> Fetus sex	− 0.36	0	− 0.36	− 0.601–− 0.077*

The 95% confidence intervals were determined by bootstrapping validation.

## Discussion

Fetal characteristics are influenced by complex biological interactions between intrauterine (e.g. nutritional exchange through the placenta, position in the uterus) and extrauterine factors (e.g. environment, physiological condition of the mother, genetic potential of the fetus)^14^. We found that gastrointestinal helminths, and particularly *M. marshalli,* were both directly and indirectly associated with lighter and smaller fetuses in Dall’s sheep. Additionally, contrary to what is typically predicted by ecological theory for dimorphic species like Dall’s sheep^28,57^, we observed that ewes in good body condition, which typically have low parasite intensity, were more likely to be carrying female than male fetuses (Fig. 2D).

Although the association of parasites with smaller fetuses is very intuitive reports in the wildlife literature are rare. The cost of parasites for wild ungulates can be substantial, particularly in species from environments marked by high seasonality or extreme seasonal environmental conditions such as Dall’s sheep, bighorn sheep, and others^58,59^. Dall’s sheep have relatively low pregnancy rates (~ 75%)^38^, compared to other wild sheep (e.g. bighorn sheep ~ 90%^60,61^), with pronounced interannual variation in timing and synchrony of parturition^41^. This may suggest that this species is particularly susceptible to costly energetic factors like parasitic infections, especially during periods of energetic stress like breeding season and pregnancy.

The gestation period for Dall’s sheep, which occurs under the extreme cold and dry conditions of the Subarctic and Arctic winter^41,42^, coincides with the seasonal peak of *M. marshalli* abundance in this species^62–64^. Here we suggest two hypotheses that could explain the association of fetus size with the intensity of parasite infection. First, *M. marshalli* may have a direct effect on the fetus by decreasing the nutrient availability for development. *Marshallagia marshalli* causes a variety of structural changes in the abomasum as a result of larval migration through the gastric glands during maturation and the mechanical damage of adult parasites attached to the mucosa^65,66^. This results in an increase in abomasal pH, and an increase in both serum pepsinogen and serum gastrin concentration, which in turn decreases nutrient metabolism in the abomasum. This effect of parasites on the metabolism of the gestating female is substantial, particularly in terms of decreased protein availability for the growing fetus^67^. These factors, together with the appetite depression typically produced by ostertagiine nematodes^68^ and the environmental constraints during winter within Dall’s sheep range, could have an additive effect on the host, limiting the resources for fetus development, resulting in smaller fetuses.

Not mutually exclusive, in our second hypothesis we suggest an indirect effect of *M. marshalli* on time of conception, as critical reproductive variables, such as the timing of ovulation, depend on the body condition of the mother^69^. Here, we make the assumption that parasite intensity of ewes in the winter (our study period) is correlated to that of the previous summer. Parasites are rarely distributed evenly within a population due to differences in exposure, immune response, and tolerance that are individually determined (through genetics and/or behaviour). This results in individuals that consistently have higher (or lower) infection intensities relative to other individuals^70,71^. Perhaps supporting a hypothesis of delayed conception, the reduced fetal size in ewes with high parasite intensity may be a consequence of parasite-induced energetic constraints and consequent later conception dates resulting in shorter development time for the fetuses of these highly parasitized mothers^25,72,73^. In our previous study, non-pregnant females from the same group of animals had significantly higher parasite infection intensities and lower body condition than pregnant females^39^. Assuming similar gestation time among fetuses, delayed time of conception can result in births occurring later in the season and, as a consequence, to a variety of post-birth costs for both the newborn and the mother. For instance, in bighorn sheep, lambs that are born later in the season have higher mortality than early-born ones, probably because of the energetic cost of a shortened access that late-lambs would have to high-quality forage or/and lactation occurring when the quality of the forage declines (e.g. low quality or insufficient milk production)^74,75^. We did not observe a direct effect of ewe body condition on fetal development. This may be because, in capital breeders like Dall’s sheep, breeding only occurs when body reserves reach a threshold condition. Heavily parasitized ewes may have taken longer during the previous fall to reach this critical threshold, yet were able to maintain this weight through gestation, thus masking any effect of body condition on fetal development.

The association of sex and maternal condition remains as a central paradigm to understanding life-history strategies and evolutionary theory. Our analyses revealed that pregnant Dall’s sheep in better condition, with fewer parasites, were more likely to have a female than a male fetus. This is consistent with the Local Resource Competition theory which suggests that, in philopatric species, mothers in poor condition will produce more sons because they often are the dispersing sex and thus less likely to compete for resources in the future^29^. This effect can be enhanced when a strong maternal transmission of condition to daughters leads to fitness maximization by producing good-quality daughters instead of average quality sons^76^. For instance, a high-quality territory can influence reproductive value (fraction of a future population that has descended from a female) more than it does immediate reproductive success (number of offspring produced in a lifetime), therefore it may be beneficial for mothers in good condition to produce a high-quality daughter who can maintain the territory and also produce more high-quality daughters in time^77–79^.

Our results are contrary to what is predicted by the Trivers and Willard hypothesis, in which case, Dall’s sheep in good condition would be expected to carry sons instead of daughters. An explanation for this may be found in the extension and generalization of the Trivers and Willard hypothesis suggested by Schindler et al.^80^. They incorporated male-specific demographic parameters commonly observed in polygynous species (e.g. high mortality rate, different sex-specific reproductive schedules, and more risk-prone life-history tactics) into two-sex modelling approaches and demonstrated that changes in these demographic parameters result in different sex allocation tactics. For instance, in a model parameterized with data from Columbian ground squirrels (*Urocitellus columbianus*) where the identical mortality rate of sexes was assigned, a reversal of the Trivers and Willard effect was predicted, with mothers in good condition producing more daughters than sons. Reversal of the Trivers and Willard effect has been observed also in bighorn sheep^81^ and several other ungulates^76^. This context-dependent framework predicts changes to the optimal sex allocation tactics over a relatively small range of demographic values highlighting the importance of the species-specific reproductive value of daughters or sons in order to determine the optimal sex allocation tactics for a mother.

Most of the evidence describing physiological mechanisms for facultative adjustment of sex ratios is related to the role of maternal glucose and stress hormones, yet the results are ambiguous and still far from consensus^82^. For instance, high glucose concentrations around conception can influence sex ratios by increasing the survival of male blastocysts through its interaction with luteinizing hormone (LH)^31,83,84^. Similarly, high-glucose-mediated mechanisms have also been associated with an increased likelihood of mothers in good condition having daughters instead of sons^32^. Although our results are aligned with the latter mechanism, as parasites, through their negative influence on host condition, can lower glucose concentrations^85^, the evidence is far from conclusive and more investigation focused on the physiological, behavioural and ecological mechanisms behind facultative adjustment of sex ratios is needed.

As a highly seasonal ungulate, Dall’s sheep are particularly vulnerable to climate warming and its effect on the phenology of many ecological processes^48,49^. While changing climatic conditions can certainly favour the development and transmission of parasites at high latitudes, they can also cause the opposite effect by, for instance, changing parasite (or host) phenology leading to a spatial and/or temporal mismatch between host and parasites^86–88^. Predicting the consequences of climate change on parasite dynamics remains a challenge as complex interactions among host, environment and the parasites need to be considered and a variety of outcomes can be expected^88,89^. Regardless of the direction of these changes in parasite dynamics, our results suggest they can have a variety of subtle consequences in Dall’s sheep population dynamics, not only through a direct influence on fetal development but also through an indirect impact on fetus sex allocation.

In this research, we have demonstrated subtle and important mechanisms through which parasites can affect host population dynamics. It is well documented that *M. marshalli* can negatively impact the fitness of wild and domestic ungulate species^39,90^. Our results suggest that this negative effect also extends to fetal development and fetal sex allocation, via a combination of direct and indirect effects of *M. marshalli* on the fetus and its mother. These results gain even more relevance in the current context of strong environmental disturbance and accelerated changes in climate, as we showed that shifts in parasite dynamics, independent of the direction of these changes, can have direct and indirect consequences on host population dynamics through impacts on fetal development and sex allocation. Refining our understanding of the impact that individual parasite species, as well as parasite communities, can have on host dynamics is critical to comprehend complex ecological processes.

## Supplementary information

Supplementary Information 1.

Supplementary Information 2.
