# Supplementary material for: Parasite intensity drives fetal development and sex allocation in a wild ungulate
Source: Sci Rep. 2020 Sep 24;10:15626. doi: 10.1038/s41598-020-72376-x (PMC7518422; doi:10.1038/s41598-020-72376-x)
Supplement: Supplementary file 1 — Supplementary Information 1. [file 41598_2020_72376_MOESM1_ESM.pdf]

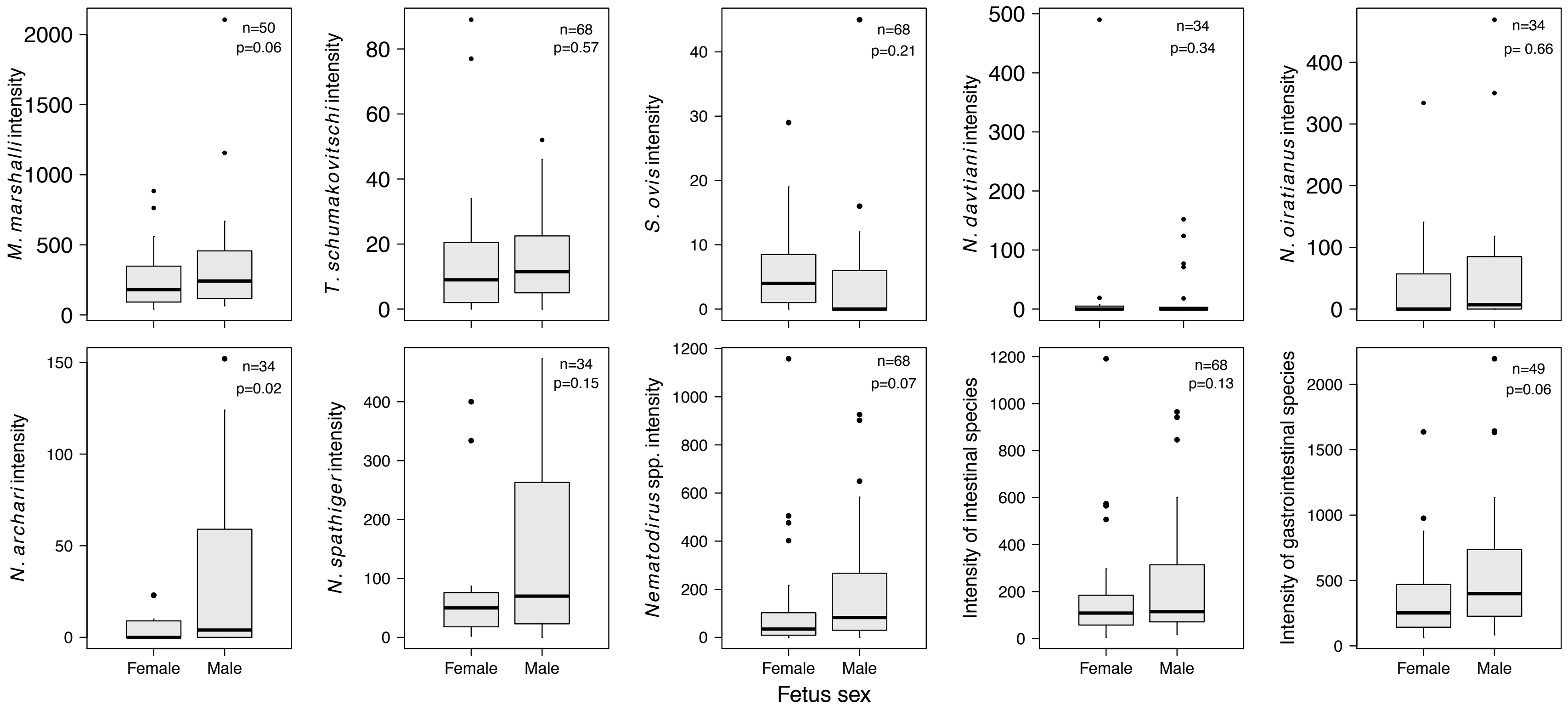

Supplementary Figure S1. Intensity of gastrointestinal helminths of pregnant Dall's sheep relative to fetal sex. The sample size (n) and p-value from Permutation test comparing the parasite species intensity in the ewe and fetal sex are indicated in the upper right corner of each figure. Figure created using R (Version 3.5.2) (R Core Team, 2013. R: A language and environment for statistical computing).
