# Supplementary material for: Parasite intensity drives fetal development and sex allocation in a wild ungulate
Source: Sci Rep. 2020 Sep 24;10:15626. doi: 10.1038/s41598-020-72376-x (PMC7518422; doi:10.1038/s41598-020-72376-x)
Supplement: Supplementary file 2 — Supplementary Information 2. [file 41598_2020_72376_MOESM2_ESM.pdf]

SUPPLEMENTARY MATERIALS

Parasite intensity drives fetal development and sex allocation in a wild ungulate

O. Alejandro Aleuy, Emmanuel Serrano, Kathreen E. Ruckstuhl, Eric P. Hoberg, and Susan Kutz.

Supplementary Table S1. Descriptive statistics of variables used to determine the association of parasite intensity and diversity in fetus development and fetus sex of Dall's sheep.

	**n**	**Median ewe age (years)**	**Median ewe weight (g)**	**SMI**	**Fetus weight (kg)**	**Fetus length (cm)**
**1971**	18	5.75<br>(2.75-11.75)	54.2<br>(48.7-63.1)	49.8<br>(43.5-58.1)	329.7<br>(574.9-157.7)	26.5<br>(32.5-22.0)
**1972**	51	6.75<br>(1.75-11.75)	49.2<br>(39.1-58.3)	47.6<br>(37.1-55.2)	202.2<br>(448.6-11.4)	23.5<br>(33.7-10.5)

Supplementary Table S2. Correlations among latent variables (LV) in the causal model for fetus development of Dall's sheep from the Mackenzie Mountains, Canada.

		Correlations with LVs			
	W	Ewe age	Ewe parasite intensity	Ewe condition	Fetus development
Ewe age					
Age in years	1	1	0.162	0.017	-0.233
Ewe parasite intensity					
M. marshalli	1	0.162	1	-0.437	-0.329
Ewe condition					
SMI	1	0.017	-0.437	1	-0.135
Fetus development					
Weight	1.482	-0.270	-0.322	0.091	0.979
Total length	-0.541	-0.308	-0.273	0.001	0.833

W=manifest variables' bootstrapped weights.

Supplementary Table S3. Correlations among latent variables (LV) in the causal model for fetus sex in pregnant Dall's sheep from the Mackenzie Mountains, Canada.

	W	Correlations with LVs		
		Ewe parasite intensity	Ewe condition	Fetus Sex
Ewe parasite intensity				
M. marshalli	1	1	-0.421	0.187
Ewe condition				
SMI	1	-0.421	1	-0.375
Fetus sex				
Fetus sex	1	0.187	0.375	1

W=manifest variables' bootstrapped weights.
